# Survival and longevity of European rulers: geographical influences and exploring potential factors, including the Mediterranean diet — a historical analysis from 1354 to the twentieth century

**DOI:** 10.1007/s11357-023-00957-5

**Published:** 2023-11-28

**Authors:** Bálint Madarász, Vince Fazekas-Pongor, Zsófia Szarvas, Mónika Fekete, János Tamás Varga, Stefano Tarantini, Anna Csiszar, Vincenzo Lionetti, Adam G. Tabák, Zoltan Ungvari, Judit Forrai

**Affiliations:** 1 Department of Public Health, Faculty of Medicine, Semmelweis University, Üllői út 26, Budapest, H-1085 Hungary; 2 Department of Pulmonology, Semmelweis University, Budapest, Hungary; 3 Vascular Cognitive Impairment and Neurodegeneration Program, Oklahoma Center for Geroscience and Healthy Brain Aging, Department of Biochemistry and Molecular Biology, University of Oklahoma Health Sciences Center, Oklahoma City, OK USA; 4 Department of Health Promotion Sciences, College of Public Health, University of Oklahoma Health Sciences Center, Oklahoma City, OK USA; 5 International Training Program in Geroscience, Doctoral School of Basic and Translational Medicine/Department of Public Health, Semmelweis University, Budapest, Hungary; 6 The Peggy and Charles Stephenson Cancer Center, University of Oklahoma Health Sciences Center, Oklahoma City, OK 73104 USA; 7 Unit of Translational Critical Care Medicine, Scuola Superiore Sant’Anna, Pisa, Italy; 8 Fondazione Toscana Gabriele Monasterio, Pisa, Italy; 9 Department of Internal Medicine and Oncology, Faculty of Medicine, Semmelweis University, Üllői út 26, Budapest, H-1085 Hungary; 10 Department of Epidemiology and Public Health, University College London, 1-19 Torrington Place, London, WC1E 6BT UK

**Keywords:** Longevity, Mediterranean diet, Aging, MedDiet, Kings, Rulers, Historic, Healthy aging

## Abstract

Significant regional variability in lifespan in Europe is influenced by environmental factors and lifestyle behaviors, including diet. This study investigates the impact of geographical region on the lifespan of European rulers spanning from the fourteenth century to the present day. By analyzing historical records and literature, we aim to identify region-specific dietary patterns and lifestyle factors that may have contributed to longer lifespans among rulers. The hypothesis to be tested is that rulers from Southern European countries, where the traditional Mediterranean diet is consumed by the local people, may exhibit longer lifespans compared to rulers from other regions, due to the well-documented health benefits associated with this dietary pattern. We extracted comprehensive information for each ruler, encompassing their sex, birth and death dates, age, age of enthronement, duration of rulership, country, and cause of death (natural vs. non-natural). To determine their nationality, we coded rulers based on their hypothetical present-day residence (2023). Utilizing the EuroVoc Geographical classification, we categorized the countries into four regions: Northern, Western, Southern, Central and Eastern Europe. While Cox regression models did not find significant differences in survival rates among regions, further analysis stratified by time periods revealed intriguing trends. Contrary to our initial predictions, the Northern region displayed better survival rates compared to the Southern region between 1354 and 1499, whereas survival rates were similar across regions from 1500 to 1749. However, after 1750, all regions, except the Southern region, exhibited significantly improved survival rates, suggesting advancements in healthcare and lifestyle factors. These findings underscore the dynamic influence of both region and time period on health and longevity. Interestingly, despite the prevalence of the Mediterranean diet in the Southern region of Europe, rulers from this region did not demonstrate longer lifespans compared to their counterparts in other regions. This suggests that additional lifestyle factors may have played a more prominent role in their longevity. In conclusion, our study sheds light on the intricate relationship between region, time period, and lifespan among European rulers. Although the Mediterranean diet is often associated with health benefits, our findings indicate that it alone may not account for differences in ruler longevity across regions. Further research is warranted to explore the impact of other lifestyle factors on the health and lifespan of European rulers throughout history.

## Introduction

Research suggests that there may be regional variation in the rate of aging, with some populations aging more slowly than others [1]. An important example is the significant lifespan variation in Europe, with some countries boasting longer life expectancies than others. While genetic factors may play a role, environmental factors such as access to healthcare, socioeconomic status, and lifestyle behaviors also have a significant impact on the rate of aging and thereby lifespan. In modern times, countries in Northern Europe, such as Iceland and Norway, tend to have longer lifespans than countries in Eastern Europe [2]. Additionally, recent studies have found that income inequality and poverty can have a significant impact on the rate of aging and lifespan, with individuals living in poverty aging more unsuccessfully and having shorter lifespans than those with higher incomes [3]. Understanding lifespan variation in Europe is crucial for developing effective public health policies and interventions aimed at improving overall health outcomes and reducing premature mortality. By identifying the factors that contribute to healthier aging and longer lifespans in the populations of certain countries, policymakers and health professionals can work to implement strategies to promote healthier behaviors and improve health outcomes in countries with shorter lifespans.

There are different methods to elucidate the mechanisms underlying lifespan variation, including genetic, epidemiological, and demographic studies. By utilizing these various approaches, researchers can gain a better understanding of the complex factors that contribute to differences in lifespan across populations. Analyzing historical data, such as birth and death records, can provide insights into the mechanisms underlying lifespan variation in Europe, such as the impact of region-specific lifestyle factors on mortality rates. Historical data can also help identify trends and patterns in lifespan over time, which can inform public health policies and interventions aimed at promoting healthy aging.

Diet is one of several lifestyle factors that may contribute to regional variation in lifespan, with certain dietary patterns, such as the Mediterranean diet, associated with a reduced risk of chronic age-associated diseases [4], including ischemic heart disease, stroke, vascular cognitive impairment, type 2 diabetes, and certain types of cancer [5]. Therefore, understanding the relationship between regional variation in diet and life expectancy can provide important insights into the health of populations in different regions.

In this study, we aim to investigate regional variation in the lifespan of European rulers throughout history by analyzing the documented longevity of kings and other rulers. Through this analysis, we hope to uncover historical trends in regional variation of lifespan and shed light on the relationship between region-specific lifestyle factors, such as regional cuisine and life expectancy. Analyzing the lifespans of rulers from different regions and time periods is a valuable tool for exploring the potential impact of diet and other region-specific lifestyle factors on lifespan because this information is often available in historical records. Birth and death records of commoners are typically not available or are incomplete, making it difficult to study the lifespan of the general population over longer time periods. Importantly, this approach also removes socioeconomic factors from the analysis. As rulers throughout history had access to the best available food and medical care, independent of their countries’ economic background, their lifespan can provide insight into the potential impact of diet on longevity, independent of other socioeconomic factors.

Our hypothesis is that rulers of Southern European countries, where the traditional Mediterranean diet is consumed, may have longer lifespans than rulers from other regions due to the well-documented health benefits of this diet [6]. The Mediterranean diet, which is rich in plant-based foods, fish, and healthy fats, has been linked to a reduced risk of chronic age-associated diseases and improved overall health outcomes, according to previous research [5–8]. In addition, we aimed to test our hypothesis that rulers of Northern countries, where diets high in saturated fats are more common and ingredients of the Mediterranean diet are not readily available, may exhibit reduced life expectancy. To explore this hypothesis, we conducted a detailed analysis of the lifespans of European rulers from different geographic regions throughout history.

## Methods

### Population

To gather our data on European rulers, we utilized various online sources, including Wikipedia. While some may question the accuracy of such sources, studies have shown that the science entries on Wikipedia are nearly as reliable as traditional sources like the Encyclopedia Britannica [9, 10]. To ensure the validity of our data, we cross-referenced our population of rulers with the dynastic tables found in Morby’s *Dynasties of the World* (2002) [11]. We began our data collection with rulers who were already in power by 1354, a year marked by the devastation of the Black Death across Europe between 1346 and 1353, which often caused rulers to succumb to infection [12]. Our data collection continued until the last ruler of a country that ceased to be a monarchy or the first ruler still alive if the country remained a monarchy. We limited our analysis to European rulers to maintain consistency in our sample population.

### Variables

**Fig. 1:**
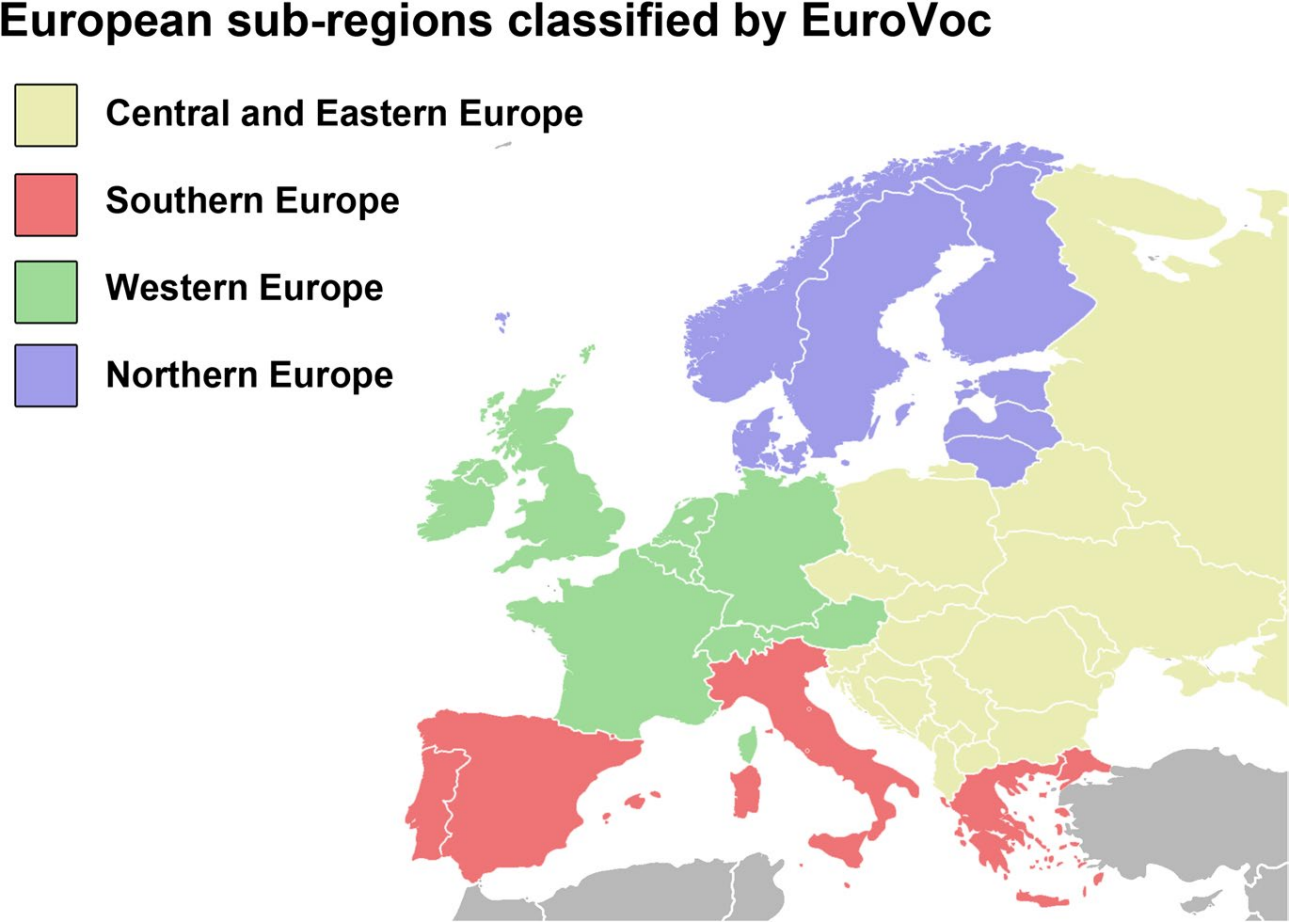
Classification of European sub-regions based on EuroVoc, with country borders representing the divisions as of 2023

We extracted various kinds of information for each ruler, including their sex, birth and death date, age, age of enthronement, duration of rulership, country, and cause of death (natural vs. non-natural death). The definition of non-natural death included the deaths caused by unexpected events, poisoning, accidents, murders, execution, or war. If we did not have any specific information regarding the cause of death, we assumed a natural death occurred. To ensure the accuracy of our data, deaths were coded separately by two investigators, and in case of disagreement, consensus was reached through discussion. We also coded the nationality of each ruler based on where they would have lived at the present day (2023). If a ruler governed in more than one country or moved to another country, their nationality was coded according to where they lived the longest. Finally, we classified the countries into the following regions based on the *EuroVoc* Geographical classification: Northern, Western, Southern, Central and Eastern Europe (as shown in Fig. 1) [13].

### Statistical analysis

**Fig. 2:**
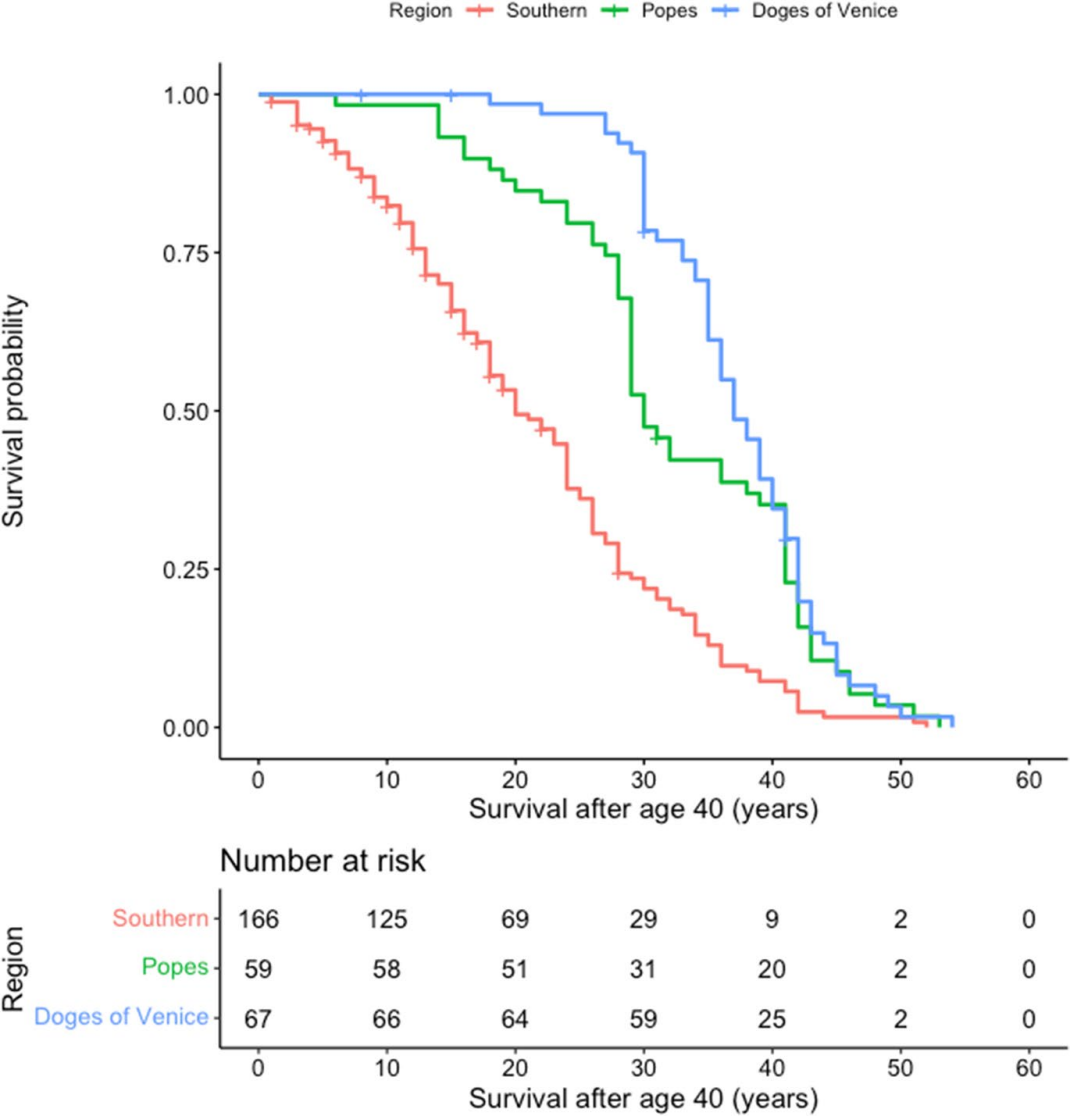
Kaplan-Meier survival curves for rulers in the Southern region, as well as for popes and Doges of Venice. The *x*-axis shows survival after age 40, while the *y*-axis displays the survival probability

Normal distribution of continuous variables was tested with Shapiro-Wilk test. As continuous variables followed a non-normal distribution, Kruskal-Wallis tests were used to compare continuous variables (age, age of enthronement, and duration of rule) among regions. Cox-regression models were created for natural vs. non-natural death as outcome and age as follow-up. Only those participants were included in the analysis who reached at least age 40, the approximate life expectancy up until the twentieth century [14, 15]. Since the Mediterranean diet is often associated with health benefits [6], we used the Southern region as reference in all our analyses. Popes (*n* = 59) and Doges of Venice (*n* = 67) were excluded from the Southern region, as they were significantly older when first elected, and thus would have led to bias. The popes and Doges of Venice stood out with a mean age of enthronement at 64 and 70, and a mean age of 70 and 77, respectively, indicating significantly better survival rates than rulers in other regions. For comparison, the mean age of enthronement and mean age for rest of the rulers in the Southern region was 27 and 57, respectively (Table 1 and Fig. 2). In the regression models, covariates included birth year and region. Birth year was centered around 1700. Linear and quadratic time terms were also introduced in the regression models. Additional analyses were conducted for three time periods: 1354–1499, 1500–1749, and 1750+. From the regression models, rate ratios (*RR*s) and 95% confidence intervals were calculated. Finally, Kaplan-Meier curves were plotted showing how survival changed over the three time periods by region, and how survival of every region compared to each other over the three time periods. Statistical significance was set at *p* < 0.05. All statistics were conducted in SPSS 24. Kaplan-Meier survival curves were constructed using R (“survminer” package).

**Table 1:** Descriptive data and Cox regression analysis for rulers of the Southern Region, popes, and Doges of Venice

	Participants *n* (%)	Age* years (*IQR)*	Age of enthronement* years (*IQR)*	Duration of rule* years (*IQR*)	Cox regression analysis RR (95% *CI*)
Southern Region	166 (56.8)	57.00 (49.75–66.00)	27.00 (18.00–37.25)	27.00 (13.00–38.00)	Ref.
Popes	59 (20.2)	70.00 (67.00–81.00)	64.00 (55.00–68.00)	9 (5.00–15.00)	0.39 (0.28–0.55)
Doges of Venice	67 (22.9)	77.00 (70.00–82.00)	70.00 (65.00–75.00)	6 (2.00–10.00)	0.27 (0.20–0.38)

*IQR* interquartile range

* Hetrogeneity *p* < 0.05

## Results

**Fig. 3:**
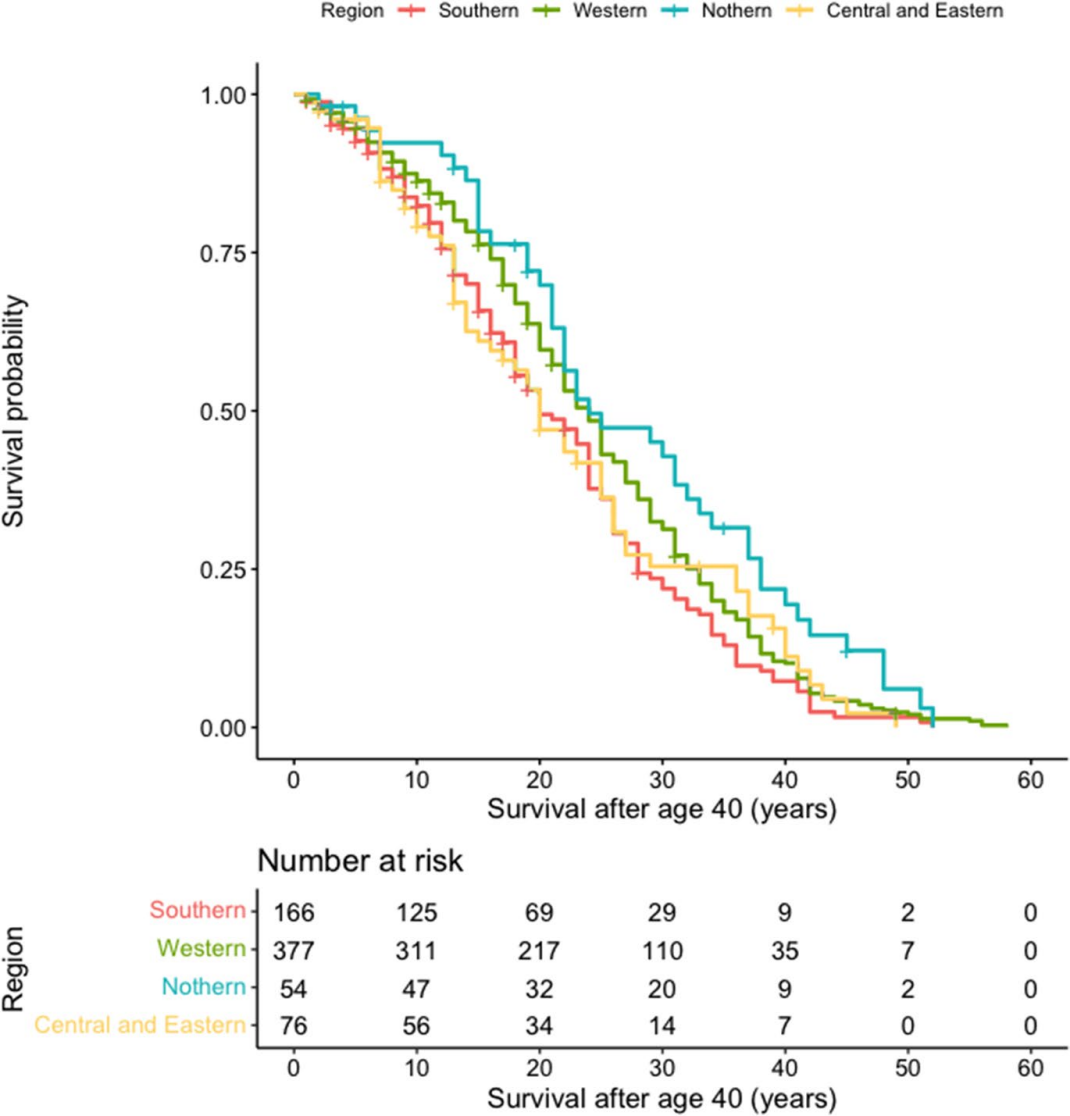
Kaplan-Meier survival curves for all rulers grouped by region. The *x*-axis represents survival after age 40, while the *y*-axis displays the probability of survival

We identified a total of 863 European rulers, excluding Popes and Doges of Venice. Of these, 190 were excluded because they died before the age of 40, resulting in a final sample of 673 rulers from the four regions: Northern (*n* = 57), Eastern, and Central European (*n* = 76), Western (*n* = 377), and Southern (*n* = 166). A total of 582 rulers died of natural causes, while 91 were recorded as non-natural death. The earliest born ruler in our sample was Luigi Gonzaga, born in 1268, and the latest ruler added was Elizabeth II, who passed away in 2022. The majority of the rulers in our sample were male (92%). We observed significant differences in age among the regions, but age of enthronement and duration of rule were similar across regions **(**Table 2). Cox regression analysis showed that hazards did not differ significantly from the Southern Region (Table 2), and these findings were supported by the Kaplan-Meier curves (Fig. 3).

**Table 2:** Descriptive data and Cox regression analysis for rulers by region

	Participants *n* (%)	Male *n* (%)	Age* years (*IQR*)	Age of enthronement years (*IQR*)	Duration of rule years (*IQR*)	Cox regression analysis *RR* (95% *CI*)
Southern Region	166 (24.7)	153 (92.2)	57.00 (49.75–66.00)	27.00 (18.00–37.25)	27.00 (13.00–38.00)	Ref.
Western Region	377 (56.0)	359 (95.2)	62.00 (53.00–71.00)	29.00 (20.00–41.00)	27.00 (16.50–39.00)	0.86 (0.70–1.06)
Northern Region	54 (8.0)	51 (94.4)	62.00 (55.00–75.50)	31.50 (23.00–45.75)	27.00 (15.00–37.00)	0.73 (0.52–1.03)
Central and Eastern Region	76 (11.3)	72 (94.7)	57.00 (49.00–66.00)	31.50 (21.00–38.75)	24.00 (13.00–37.00)	1.07 (0.77–1.46)

*IQR*: interquartile range

* Hetrogeneity *p* < 0.05

**Fig. 4:**
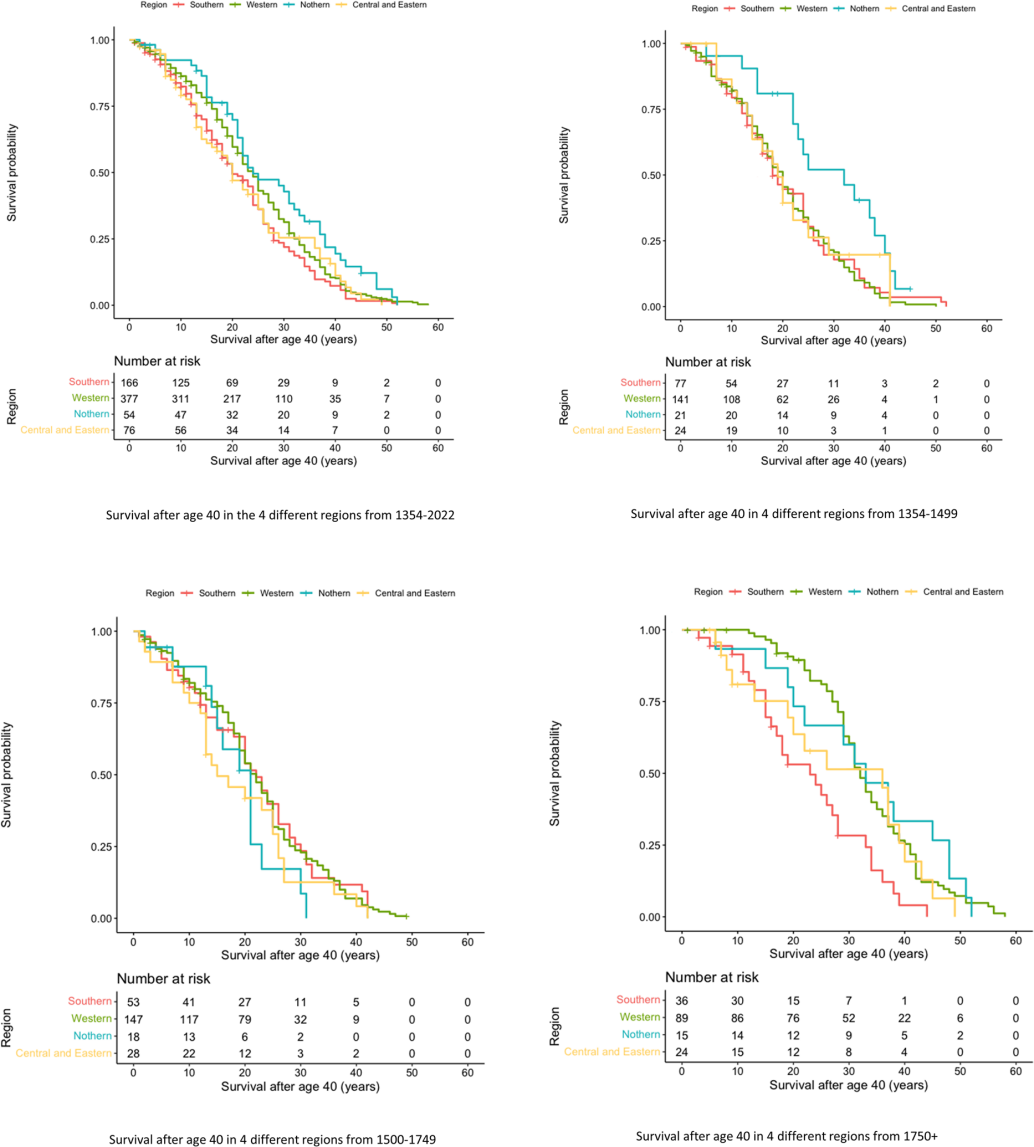
Comparison of Kaplan-Meier survival curves for regions by time periods

**Fig. 5:**
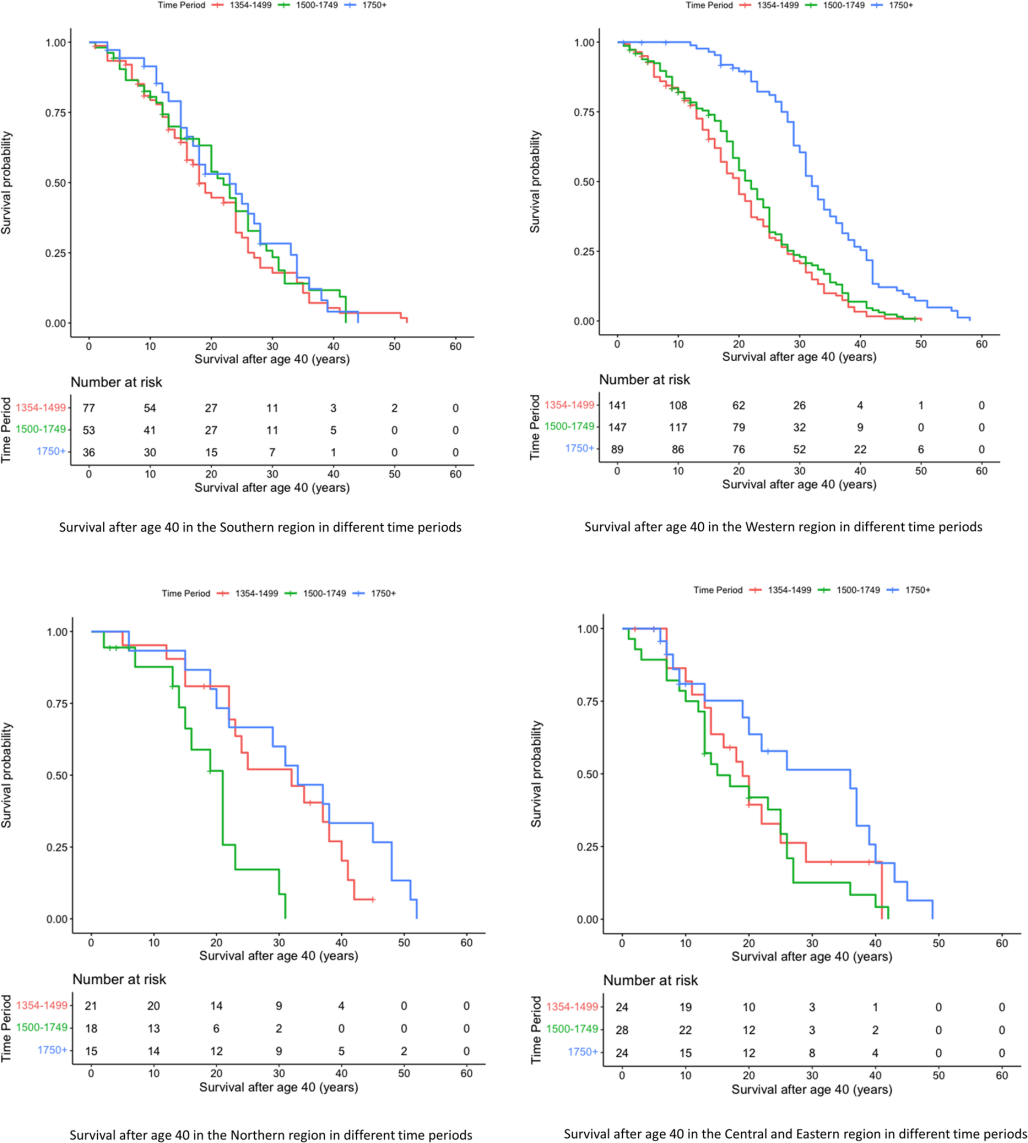
Changes in survival rates for each region over three distinct time periods. Each line represents a different region, and the *x*-axis shows the timeline. The *y*-axis displays the survival rate, indicating the proportion of rulers who survived until a specific age. The three time periods are: 1354–1499, 1500–1749, and after 1750. The graph provides a visual representation of how survival rates have changed over time in each region and how the regions compare to one another

When we stratified our analysis by time periods, we found that age of rulers and age of enthronement were similar across regions from 1354 to 1749, but differed significantly after 1750. Duration of rule was similar across all three time periods. Within the Western and Northern regions, age of rulers and age of enthronement significantly increased over time, while they remained stable in the Southern and Central and Eastern European regions. Duration of rule significantly decreased over time in the Southern and Western regions but remained stable in the other two regions **(**Table 3). Cox regression analysis showed that from 1354 to 1499, the Northern region had significantly better survival compared to the Southern region (*RR*: 0.48; 95% *CI*: 0.28–0.84), but survival did not differ significantly from the reference between 1500 and 1749. After 1750, the Western (*RR*: 0.44; 95% *CI*: 0.28–0.68), Northern (*RR*: 0.39; 95% *CI*: 0.20–0.76), and Central and Eastern European (*RR*: 0.52; 95% *CI*: 0.28-0.98) regions all exhibited better survival than the Southern region (Table 4). These findings were supported by the Kaplan-Meier curves (Fig. 4). When we examined each region separately, we found that survival of rulers in the Southern region was stable over all three time periods. In the Northern region, survival initially declined between 1500 and 1749 and then increased, while in both the Western and Central and Eastern European regions, survival increased prominently only after 1750 (Fig. 5).

**Table 3:** Descriptive statistics by EUROVOC regions through different periods

Age, years (*IQR*)				
	1354–1499	1500–1749	1750+	Heterogeneity *p*-value
Southern Region	56.00 (49.00–64.00)	60.00 (50.00–68.00)	58.00 (51.25–68.00)	0.399
Western Region	57.00 (50.00–66.50)	60.00 (51.00–68.00)	71.00 (65.50–79.50)	< 0.001
Northern Region	64.00 (58.00–77.50)	55.50 (46.75–61.00)	73.00 (60.00–88.00)	0.001
Central and Eastern Region	57.50 (50.25–61.50)	54.50 (50.50–65.75)	59.50 (47.25–77.00)	0.855
Hetrogeneity p-value	0.013	0.191	<0.001	
Age of enthronement, years (*IQR*)				
	1354–1499	1500–1749	1750+	Heterogeneity *p*-value
Southern Region	29.00 (18.00–38.50)	25.00 (17.50–37.50)	26.50 (19.25–37.25)	0.731
Western Region	25.00 (17.50–34.00)	27.00 (18.00–42.00)	40.00 (28.00–49.00)	< 0.001
Northern Region	30.00 (17.50–46.00)	26.00 (17.00–31.25)	45.00 (40.00–53.00)	0.003
Central and Eastern Region	23.00 (16.50–37.75)	32.50 (22.25–40.50)	32.00 (22.50–38.50)	0.242
Hetrogeneity p-value	0.377	0.530	<0.001	
Duration of rule, years (*IQR*)				
	1354–1499	1500–1749	1750+	Heterogeneity *p*-value
Southern Region	29.00 (13.50–37.50)	33.00 (17.00–43.00)	18.50 (6.00–29.75)	0.006
Western Region	30.00 (21.00–39.00)	29.00 (16.00–43.00)	22.00 (12.50–33.00)	0.009
Northern Region	33.00 (14.00–39.50)	23.00 (19.00–32.50)	25.00 (15.00–35.00)	0.504
Central and Eastern Region	34.00 (19.50–40.00)	23.00 (10.25–36.25)	21.50 (11.50–26.00)	0.122
Hetrogeneity *p*-value	0.381	0.338	0.338	

*IQR*: interquartile range

**Table 4:** Result of the Cox regression models by different periods and regions

	Participants *n* (%)	Male *n* (%)	Cox regression analysis *RR* (95% *CI*)
1354–1499			
Southern Region	77 (29.3)	68 (88.3)	Ref.
Western Region	141 (53.6)	135 (95.7)	0.96 (0.70–1.31)
Northern Region	21 (8.0)	20 (95.2)	0.48 (0.28–0.84)
Central and Eastern Region	24 (9.1)	24 (100)	0.83 (0.48–1.43)
1500–1749			
Southern Region	53 (21.5)	51 (96.2)	Ref.
Western Region	147 (59.8)	141 (95.9)	1.06 (0.75–1.49)
Northern Region	18 (7.3)	16 (88.9)	1.45 (0.77–2.72)
Central and Eastern Region	28 (11.4)	24 (85.7)	1.53 (0.94–2.51)
1750+			
Southern Region	36 (22.0)	34 (94.4)	Ref.
Western Region	89 (54.3)	83 (93.3)	0.44 (0.28–0.68)
Northern Region	15 (9.1)	15 (100)	0.39 (0.20–0.76)
Central and Eastern Region	24 (14.6)	24 (100)	0.52 (0.28–0.98)

95% *CI:* 95% confidence interval, *RR:* risk ratio

## Discussion

The results of our analysis provide insight into the survival rates of European rulers across different regions and time periods. While our Cox regression models did not find significant differences in survival rates among regions, further analysis stratified by time periods revealed interesting trends. Contrary to our prediction, the Northern region exhibited significantly better survival rates compared to the Southern region between 1354 and 1499. In contrast, survival rates were similar among regions between 1500 and 1749, indicating that health and longevity may have become more evenly distributed across Europe during that time. However, after 1750, all regions except the Southern region exhibited significantly better survival rates, suggesting possible improvements in healthcare and lifestyle factors that benefited European rulers in those regions. These findings are further supported by the Kaplan-Meier curves, which illustrate how survival rates varied across regions and time periods. The relatively high survival rates of the Northern region between 1354 and 1499 followed by a decline between 1500 and 1749 and subsequent increase after 1750 is particularly noteworthy, as it illustrates that regional differences in health and longevity of elites can be dynamic and subject to change over time. Overall, our findings highlighted the influence of region and time period on health and longevity. The results of our investigation extend the findings of previous studies examining the lifespan of European royalty, indicating a significant increase in life expectancy from 800 to 1800, independent of the decline in violent deaths [16]. Interestingly, these studies revealed a gradient in the lifespan of the European elite society, with longer lifespans observed from south to north and from east to west [16].

These gradients partially still exist today; however, the east-to-west gradient is stronger than the south-to-north one for life expectancy at birth [2]. The highest life expectancy at birth in Europe (2020) is observed in Norway (83.3 years) and Switzerland (83.1), while the lowest is found in Romania (74.2) and Bulgaria (73.6) [17]. If we examine smaller geographic regions, the highest life expectancy can be observed in French Corsica (84), the Balearic Islands in Spain (83,9), and in the Epirus region of Greece (83.8), while the lowest is found in the North-West Region of Bulgaria (72.1) [17].

Some of these present-day differences in longevity can be explained by the economic differences, and one of the indicators of these developments is the gross domestic product (GDP) value of the different countries. GDP shows a strong correlation with life expectancy [18]. In 2020, Norway had the highest GDP per capita (68,850 USD) followed by Switzerland (60,040 USD), whereas the lowest was found in Albania (3810 USD) and North Macedonia (4100 USD) [19]. Socioeconomic differences and inequalities in Europe affect the health of the population directly [20]. However, it is interesting to note that the correlation between GDP and life expectancy is not linear. For example, in both Greece and the Netherlands, the life expectancy at birth approximates 81.4 years, while the GDP per capita of the Netherlands is twice as high as it is in Greece [2, 19]. The same is true for Germany and Portugal too [2, 19].

Apart from GDP, lifestyle factors, such as smoking, physical activity, and diet, also influence longevity and may be responsible for the regional differences in life expectancy. In modern times, smoking is one of the most important health-related risk factors that also negatively impacts longevity [21]. According to the Eurostat in 2019, the countries with the highest proportion of daily smokers were Bulgaria (28.7%), Greece (23.6%), Latvia (22.1%), Germany (21.9%), and Croatia (21.8%). Meanwhile, the lowest smoking prevalence was observed in Sweden (6.4%), Finland (9,9%), Luxembourg (10.5%), and Portugal (11.5 %) [22]. Physical activity also plays a pivotal role in healthy lifestyle. In 2022, according to a survey conducted by the European Commission, the physically most active countries were Finland (71%), Luxemburg (63%), and the Netherlands (60%), whereas the highest levels of inactivity were found in Portugal (73%), Greece (68%), and Poland (65%) [23]. In terms of diet, in 2019 the highest fruit and vegetable consumption rates were observed in Italy, Spain, Belgium, and Ireland (more than 75%), while the lowest was found in Latvia and Romania (< 50%) [24].

As we can see from the data, northwest Europe is generally better off in terms of longevity. However, the South, for instance Greece, is also competing with the North and West even with its lower GDP per capita, higher rates of smoking, and less frequent physical activity. In our opinion, this may be partially explained by dietary factors like the Mediterranean diet of the region. This is why we hypothesized that regional variation in diet, particularly the consumption of a Mediterranean diet, is a major determinant of lifespan variation between different European countries based on previous research showing the positive impact of this dietary pattern on health outcomes and the reduced risk of chronic age-related diseases [5].

The Mediterranean diet is a dietary pattern that is traditionally followed in countries located along the Mediterranean Sea, such as Spain, Italy, and Greece in Europe as well as Turkey, Egypt, Tunisia, Morocco, and others [5]. However, it is important to note that there are variations in the specific foods consumed in each country depending on cultural, religious, and economic factors [5]. The Mediterranean diet is characterized by high consumption of fruits, vegetables, whole grains, legumes, nuts, and olive oil, moderate consumption of fish and seafood, low to moderate consumption of dairy products, and low consumption of red meat and processed foods [6]. The Mediterranean diet has been widely studied for its potential anti-aging health benefits, including a reduced risk of chronic age-associated diseases [5, 7] and improved longevity in Greece, Italy, and in the Mediterranean region [5, 7]. Several studies have found that adherence to the Mediterranean diet is associated with a reduced risk of mortality from cardiovascular disease and cancer [5–8]. There is growing evidence that individual components of the Mediterranean diet exert multifaceted anti-aging effects both in pre-clinical studies and clinical investigations. Resveratrol is a polyphenolic compound that is found in red wine, grapes, and other berries. It has been shown to have anti-inflammatory, antioxidant, and anti-cancer properties [25–41] and may also promote longevity by activating certain enzymes and transcription factors (i.e., SIRT-1 [42], and Nrf2 [43, 44]) that are involved in the regulation of cellular and molecular processes of aging. In addition to resveratrol, several other compounds present in the Mediterranean diet have been shown to exert significant anti-aging effects. One such compound is hydroxytyrosol [45–48], which is found in high concentrations in extra virgin olive oil. Hydroxytyrosol has been found to exert strong antioxidant effects, which may help to protect against oxidative stress and inflammation. Additionally, flavonoids such as quercetin, which is found in onions, apples, berries, citrus fruits, leafy green vegetables, tomatoes, and broccoli, have also been shown to possess potent antioxidant and anti-inflammatory effects, potentially contributing to the anti-aging effects of the Mediterranean diet [49–51].

In this study, we aimed to explore the potential impact of the Mediterranean diet and geographic location on the lifespan of European rulers. By examining the dietary patterns of European rulers from different geographic regions, we hoped to shed light on the potential impact of the Mediterranean diet on longevity and gain a better understanding of the factors that contribute to overall lifespan. Throughout history, the diets of royals have been the subject of fascination and curiosity, as they often reflect not only personal tastes but also cultural and societal norms. In the following sections, we will explore what is known about the royal diets through the centuries, from the medieval times to modern day, and the factors that have influenced their dietary patterns.

### Before the New World: 1354–1499

The evolution of nutrition over the centuries has been significant, with royalty having access to the finest ingredients and rarely experiencing hunger [52]. Meals were not only about satisfying hunger but also had social significance for the nobility. A well-laid table, the number of courses, and the composition of the dishes were all regulated by court etiquette. The abundance of food served as an expression of wealth, prosperity, and power, while the feast itself established a clear social hierarchy [52, 53].

The cuisine of a given region is influenced by various factors, including climate and geographic location. For instance, Southern Europe’s natural geography limits food production to lowlands and hilly areas, resulting in a shortage of basic foodstuffs such as cereals but abundant seafood [54]. Near the Mediterranean Sea, viticulture and cultivation of olives were important. In contrast, Central Europe’s temperate climate favored cereal production and animal husbandry [55]. In Western Europe, animal husbandry was a mainstay of the agricultural economy [56]. Barley and wheat were the most important crops in most European regions; oats and rye were also grown, along with a variety of vegetables and fruits. In Iberia, the Arab Agricultural Revolution introduced a large number of new crops, which continued to be cultivated, including sugar cane, rice, citrus, and figs [57]. In the North, agriculture was less sustainable, with scarce arable land mainly used for barley and millet and pastures mainly used for cattle and sheep [58], while animal husbandry, hunting, and fishing were also practiced [59]. Vegetables such as cabbages, onions, peas, and beans were widely cultivated in Europe. In comparison to the Mediterranean region [60], where the dry summer weather limited the availability of animal feed, livestock held greater significance in Northern Europe [61]. The primary farm animals in the Mediterranean were sheep and goats, while in Northern Europe, both cattle and pigs played significant roles.

The economic circumstances played a significant role in shaping the culture and cuisine of these regions. Following the devastating impact of the Black Death between 1346 and 1353, which claimed the lives of at least one-third of Europe’s population, there was a notable shift in food availability [12]. The reduced population meant that the same amount of natural resources was distributed among fewer individuals, resulting in a surplus of food [62]. As a consequence, a wider range of meats became accessible to a larger segment of the population until the sixteenth century, when population growth led to bread once again becoming the dominant component of meals [62]. Another crucial element to consider in the development of regional cuisines is the primary source of fat, which varies based on climate and culture. In different European regions, the usage of oil and butter (or fat) as cooking fats, along with the consumption of beer and wine, played an important role in shaping the culinary traditions. These regional variations reflect the unique agricultural practices and food preferences that emerged in response to the geographical and cultural contexts of each area.

Contrary to our initial prediction, our study found that rulers from regions where the Mediterranean diet was prevalent did not live longer than those from other regions. The underlying reasons for this are likely to be multifaceted and may involve the differences between the dietary habits of the general population and the ruling elite and a complex interplay of genetic, environmental, and lifestyle factors.

While climate and geography do impact the availability and variety of food and the prevalence of the consumption of Mediterranean diets, the cuisine enjoyed by European nobility may have remained relatively consistent throughout the ages in accordance with the fashions of the time and the support of the trade. The ability to consume copious amounts of food and drink was viewed as a demonstration of strength and valor, with meat being a particularly prized ingredient [63]. It was not uncommon for whole roasted animals to be served and carved at the table immediately before consumption [64]. Additionally, smaller animals like lambs and rabbits, or other ingredients like eggs, mushrooms, and vegetables, were often placed inside larger animals like cattle, deer, or pigs and cooked whole [64]. Over time, new ingredients and dishes were introduced, such as sugar and later colonial goods. The quality and quantity of food were indicators of social status and wielded as tools of power and diplomacy, along with the indulgence of pleasures.

The European Middle Age kitchen was greatly influenced by the Roman Empire. The Roman kitchen is relatively well-known thanks to the ancient recipes published by Apicius [65]. The diet of the time contained ingredients, such as fish, wine, herbs, spices, pork, poultry, and bread made with sourdough [65]. Popular vegetables were Brussels sprout, beets, lettuce, onions, cucumbers, asparagus, while commonly consumed fruits were peaches, grapes, apples along with nuts and sweet chestnuts [65].

To gain insight into the diet of the nobility, reports from feasts and cookbooks with recipes of the upper classes provide the best sources [66]. In one of the first cookbooks after antiquity, *The Book for Cook* (*Libro per cuoco*) by an unknown author of the mid-1300s, it was evident that Venice was the main hub of the European spice trade, and the nobility there had easy access to a wide variety of luxury spices [66]. For example, recipe LXXIII in *The Book for Cook* is a mixture of pepper, cinnamon, ginger, cloves, and saffron that was used as a pre-prepared spice mixture ingredient for other recipes [67]. The high consumption of spices and herbs may be explained by two factors. First, they were expensive and a symbol of social status, and second, they were also consumed as a medicine based on the humoral pathological concept of the time [66, 68]. The recipe collections of the fourteenth and fifteenth centuries in Western Europe and Italy were very similar to each other because of the culture of adding spices to everything [66]. The nobility consumed vast amounts of meat seasoned with imported spices along with white bread and delicacies, such as almonds, dates, and luxury products [66].

While there were likely some notable differences in dietary patterns between regions, other factors such as physical activity, high levels of stress from political, personal, or societal pressures, excessive alcohol use and even genetics (e.g., royal families with significant inbreeding) may have had a greater impact on lifespan. These findings highlight the complex interplay between diet and other factors that contribute to overall health and longevity. A possible explanation for the significantly better survival observed for the Northern region — could be that Nordic countries were possibly less engaged in military activities than the other regions in this period. The lower number of conflicts and higher economic stability (e.g., founding of the Kalmar union in 1397 [69]) could have granted a better quality of life for the rulers of the Northern region [70]. Another factor that may explain the better survival of rulers in the Northern region is the lack of malaria at higher altitudes [71, 72], as our database indicates that only Southern and Western rulers were documented to have died of malaria during this time period. Other possible explanations may be linked to infectious diseases. The Scandinavian region was one of the most severely affected region by the Black Death plague that ended in 1353 [73]. The decrease of population resulted in a relative prosperity and an increase in food availability in the coming years [62]. However, it is less likely that this would have directly affected the survival of the rulers in this region, who always had access to sufficient amounts of food regardless of the prosperity of the general population.

### The age of exploration and geographic discoveries — transformations in a Brave New World: 1500–1749

Contrary to our prediction, survival rates were similar among regions during the period of exploration and great geographic discoveries from 1500 to 1749. Notably, the Northern rulers’ advantage observed in the previous period disappeared. There could be various reasons for this, including changes in dietary habits, as well as geopolitical changes, conflicts, and weather conditions. For instance, the Northern countries engaged in a higher number of conflicts than before, which could have had a detrimental effect on their economy after the dissolution of the Kalmar Union in 1523 [69]. The Thirty Years’ War had a profound impact on Europe, both in terms of loss of life and destruction of property. Moreover, the Little Ice Age period from the middle of the sixteenth century to the middle of the nineteenth century resulted in a colder climate in the Northern Hemisphere [74]. This led to famine and the death of approximately one-quarter to one-third of the population of Finland in 1696–1697 [62, 75].

During this period, significant changes occurred in the world of cuisine; feasts were still a tool of power and a platform to meet and build agreements. For instance, Cristofor da Messibugo, the steward of the Duke of Ferrara, documented the four courses of the banquet held by the Este family in Ferrara in 1529, which consisted of a wide variety of meat, fish, pastry, fruits, and spices [66, 76]. John Dickie calculated that if the 104 guests ate an equal share of the courses, they would have consumed an astonishing amount of food, including eighteen portions of eleven different fish, three whole birds the size of capons, another five smaller birds, three portions of meat, and four portions of sausage, salami or ham, fifteen small pastries, plus assorted blancmanges, fritters, and salads [66]. The eating habits of influential families like the Spinola family from Genoa were also studied. Frank Spooner compared their eating habits to those of the local hospital and found that while the hospital’s diet was primarily cereal-based (81% of calories were from cereals compared to 53% in the diet of the Spinola family), the Spinola family consumed twice as much meat and three times as much dairy products [62, 77]. Herb and spice consumption began to decline among the nobility during this time period, as spices like black pepper became more accessible and no longer symbolized status. Instead, tea, coffee, tobacco, chocolate, and liquors became the new luxury items. Additionally, a variety of new vegetables and fruits such as artichoke, green peas, green beans, green pepper, and melon started appearing on the rulers’ tables [62]. The explorations and the agricultural revolution also introduced potato, tomato, and corn to meals in the nineteenth and eighteenth centuries [66].

### The Industrial Epoch — is knowledge power? After 1750

Our initial prediction was also contradicted by our findings that showed a clear trend of increasing lifespan among rulers in Northern and Western regions after 1750, while the Southern region did not experience a similar improvement in life expectancy. This discrepancy in lifespan trends between regions highlights the need for further investigation into the complex interplay of socioeconomic, environmental, and lifestyle factors that may have contributed to these differences. The striking increases in the lifespan of rulers in the Northern and Western regions after 1750 are particularly noteworthy. In this time period, the Industrial Revolution, which originated in Great Britain, brought several technological advancements that boosted the economy and quality of life in most regions [78]. However, since the Southern region was the last to experience the industrial revolution’s effects, its economy and quality of life may have been adversely affected [79]. The period after 1750 also saw significant advancements in the field of medicine. The discovery of vaccination by Edward Jenner in 1796 was a major breakthrough in the prevention of infectious diseases [80]. The development of anesthesia in the mid-nineteenth century revolutionized surgery and made complex surgical procedures possible [81]. In addition, the discovery of the germ theory by Louis Pasteur in the late nineteenth century paved the way for the development of antibiotics, which have saved countless lives [82]. During the eighteenth and nineteenth centuries, countries in the Western region, including the predecessors of modern France and Germany, the Habsburg Empire and the UK were at the forefront of medical research and practice. It is possible that the advancements in medicine in the Western region during this time period played a role in the longer lifespans of rulers in these areas, as improved medical knowledge and practices may have led to better healthcare for rulers in these societies.

Diet also changed significantly in this time period. From 1600 to 1800, spices continued losing their prominence due to colonization, leading to the emergence of more distinct national culinary traditions [66]. However, the royal taste across Europe remained still quite similar during this time, as noted by John Dickie [66]. Danielle De Vooght argues that “fashions spread from court to court through their continuing contact with each other” [83], supporting the claim that the courts’ food choices were likely homogenous and characterized by excessive consumption of meat, luxurious items, and alcohol. In the eighteenth century, there was a notable disparity between economic development and population growth, leading to occasional food scarcity and starvation among the masses. It took time for the production and consumption ratios to align once again. However, with the advancement of animal husbandry and agriculture, the elimination of fallow land, the implementation of crop rotation involving cereals and legumes, and the introduction of fertilization techniques, along with the spread of new high-yield crops such as rice, maize, and potatoes, a potential famine crisis was averted. Nevertheless, while food supply became more secure, it also became increasingly one-sided and socially differentiated [62]. The diet of the poor became simpler and more limited, with wheat and meat becoming less accessible and being replaced by corn and potatoes. The growth of the population had a negative impact on nutritional culture until the mid-nineteenth century. Initially, the diet of the poor primarily revolved around cereals, but gradually, both quantitative and qualitative changes occurred. The introduction of new cooling and canning techniques towards the end of the nineteenth century, along with improved milling processes that produced cleaner wheat, made white bread more widely available to a larger segment of the population [62]. Technological progress eventually facilitated greater access to meat and bread, resulting in a better and more secure food supply for common people, but the nobility continued to demand more sophisticated and elaborate dishes, turning the dining table into a spectacle focused on representation [62]. Although the economic processes had a limited direct influence on the diet of the nobility, they indirectly impacted the eating habits and supplies found on the tables of nobles across different regions.

Another factor that could contribute to the lower survival rates in the Southern region is the prevalence of poisonings. This region has a long history of poisonings, dating back to the Roman Empire [84–86]. Our database indicates that 18 Southern rulers were potentially assassinated by poison, compared to seven Western and four Central European and Eastern rulers, with the possibility that some “natural” deaths were actually caused by hidden poisons.

The last factor that could contribute to the lower survival rates of Southern rulers is the outbreak of infectious diseases. According to Giulio Alfani, the Italian peninsula in the seventeenth century was more susceptible to infectious diseases than other regions [87]. Although outbreaks began decreasing in the eighteenth century Europe [87], they could still have had an impact on the survival rates of royalties both directly and indirectly, by reducing the availability and quality of food and overall quality of life [62]. However, the nobility was often fortunate enough to isolate themselves from outbreaks by retreating to the countryside [88].

European rulers led a markedly distinct lifestyle from that of common folk. Still, they represent a relatively homogenous group who had access to the highest quality and quantity of food and quality health care and are protected from causes of death, such as starvation, war, and infectious diseases related to lack of hygiene and overcrowding, that often decimated the general population. Rulers were probably more similar to each other than the populations they ruled over who may have differed greatly by geographic region and political situation enabling a somewhat more precise estimation of lifespan by geography without having access to potential confounders that may have biased our results if we would have examined the general population without the adjustment for these confounders.

### Limitation

One major limitation of our study is the inability to eliminate the influence of immortal time bias. We considered age as the underlying time and only included rulers who were crowned, which meant that rulers were effectively immortal until their coronation. As a result, rulers who were crowned later in life had a distinct advantage over those crowned at a younger age since they had already survived for a longer time and were more likely to live to an older age. A further limitation of our study may be the arbitrary selection of large regions that do not necessarily represent uniform cuisines. Cuisines could differ from country to country within the same region, which may have influenced our results. Moreover, we had no information on the specific diet of individual rulers and whether the Southern rulers truly adapted the Mediterranean diet. A subsequent limiting factor is the accuracy of historical records leading to possible misclassifications for the cause of death. Another limitation is the lack of access to possible confounding factors that can significantly impact lifespan, such as smoking, alcohol consumption, or diseases. Furthermore, we did not take into consideration genetic relations either, which may also play an important factor in longevity, because the common dynastic intermarriages, frequent location changes, and consanguineous marriages make it hard to clearly account for these factors. From a statistical point of view, the Northern and Central and Eastern regions had significantly fewer rulers leading to statistical power issues. We were also not able to examine male and female rulers separately because of the small number of female rulers overall, which may have led to statistical power issues. Finally, our results are not generalizable to the whole population either, as our sample consists of highly privileged individuals that do not reflect the lifestyle of common folks, and the differences observed in our study may be probably contributed to other factors other than diet that we were not able to identify due to lack of information on these variables.

## Conclusion

The findings of our study suggest that the lifespan of Southern European rulers was not longer than that of their counterparts in other regions. In fact, after 1750, life expectancy in this area may have been even lower. Our results partially support those of a previous study that found a gradient of longer lifespans for the European elite society from South to North and East to West between 800 and 1800 [16]. However, it should be noted that our regions were quite large, which may have made it difficult to identify subtle differences. Furthermore, life expectancy is influenced by numerous factors apart from nutrition, and it is possible that our results were affected by other variables, such as geopolitical conflicts, outbreaks of infectious diseases, changing residency, regional variability in the level of medical care or the gradual expansion of the Industrial Revolution. It is also important to note that the rulers in Southern Europe may not have followed the Mediterranean diet that has been linked to a lower risk of cardiovascular disease and other chronic illnesses not to mention the possible lacking important socio-cultural aspect of the diet too [7, 8]. Common people were more likely to follow this diet, characterized by high consumption of plant-based foods such as herbs, vegetables, fruits, and pulses, as well as low consumption of red meat and also lived a physically more active lifestyle [6, 7, 66, 89]. In contrast, the cuisine of the courts may have been more similar to each other than different [66, 83], and rulers had access to a wide range of foods that may not have been available to the general population. Therefore, the dietary habits of the rulers in our study may not have been representative of the Mediterranean diet. Other factors, such as protection against physical assault and infectious diseases, economic stability, and access to the best healthcare of the time, may have also played a role in the overall longevity of European rulers. It is possible that these factors offset any differences in diet between the regions and contributed to the lack of apparent differences in our study.

## Data Availability

Data is available upon request.
